# Egocentric social networks, lifestyle behaviors, and body size in the Asian Community Health Initiative (CHI) cohort

**DOI:** 10.1371/journal.pone.0232239

**Published:** 2020-05-06

**Authors:** Candyce H. Kroenke, Gem M. Le, Shannon M. Conroy, Alison J. Canchola, Salma Shariff-Marco, Scarlett Lin Gomez

**Affiliations:** 1 Division of Research, Kaiser Permanente Northern California, Oakland, CA, United States of America; 2 Division of General Internal Medicine, Department of Medicine, UCSF Center for Vulnerable Populations, UCSF, San Francisco, CA, United States of America; 3 Department of Epidemiology and Biostatistics, University of California San Francisco, San Francisco, CA, United States of America; 4 Helen Diller Family Comprehensive Cancer Center, San Francisco, CA, United States of America; Rutgers The State University of New Jersey, UNITED STATES

## Abstract

**Background:**

Social networks have been shown to influence lifestyle behaviors in non-Latinx white (NLW) populations. We examined their influence in Asian American, Native Hawaiian and Pacific Islander (AANHPI) women.

**Methods:**

We included 477 AANHPI women from the Asian Community Health Initiative Study who provided egocentric (degree, density, composition) and epidemiologic (size, types of ties) social network data and data on alcohol intake, physical activity, smoking, diet, and body size. We used logistic regression to evaluate associations of social network measures and dichotomous outcomes, and linear regression for continuous outcomes.

**Results:**

In multivariable-adjusted analyses, higher degree and/or proportion of friends were significantly related to higher Western diet, higher odds of any alcohol consumption, and lower odds of physical inactivity and body mass index (BMI)≥23 kg/m^2^. Additionally, a higher proportion of NLW in women’s networks was related to lower Asian diet but also lower waist size. Community participation was related to higher Western diet and lower Asian diet. By contrast, degree and/or proportion of relatives were positively related to BMI, waist size and to a higher odds of BMI≥23 kg/m^2^ and of ever smoking 100 cigarettes. Being married was related to fewer alcoholic drinks per week and higher Asian diet. A higher density of relationships with frequent contact was also associated with higher Asian diet.

**Conclusions:**

AANHPI women with larger proportions of friends and NLWs in their networks had more Western health behaviors and smaller body size. Norms for health behaviors and body size may be influenced by the size, composition, and structure of social networks, relevant to chronic disease prevention.

## Introduction

In the general population, 70–90% of cancers [1] and cardiovascular and metabolic diseases [2] are due to modifiable [3] behavioral and environmental factors. High consumption of a Western diet, alcohol intake, smoking, physical inactivity, overweight, and high waist size are shared risk factors for cancer, diabetes, and/or cardiovascular disease [4–7]. However, while lifestyle behaviors are often conceptualized at the individual level, they may be strongly influenced by social networks, the webs of relationships around people.

Social network measures including size, degree (number of connections between a node and alters in an egocentric network), density (proportion of those in a network who are ties (connections), types of social ties, diversity (number of different types of social ties), and composition, have been related to lifestyle behaviors in the general population and in chronic disease populations [8–12]. Low social network diversity, defined as the variety of social ties or roles, has been related to alcohol dependency, smoking, low levels of physical activity [8, 9] and poorer health [10]. In a Swiss sample, participants with a higher proportion of exercisers in their network had higher levels of physical activity [11]. In the After Breast Cancer Pooling Project, social network variables such as social network size, social network diversity, and specific social ties were related to lifestyle behaviors in women diagnosed with breast cancer [12].

Though social networks have been implicated in the shaping and transmission of lifestyle behaviors, including diet and obesity [13, 14], little direct research in this area has been conducted in immigrants and populations other than non-Latinx Whites (NLW), a critical omission given that lifestyle behaviors of immigrants often change substantially as a function of acculturation and exposure to Western behavioral norms [15]. Given the lack of prior work in this area, we conducted exploratory secondary data analysis of data from the Asian Community Health Initiative (CHI) study and evaluated associations of social network measures on lifestyle behaviors in a sample of 477 Asian American, Native Hawaiian, and Pacific Islander (AANHPI) women, two-thirds who were immigrants to the U.S., important given that AANHPIs are the fastest growing racial/ethnic group in the U.S. [16]

## Methods

### The Community Health Initiative (CHI) study sample

The Asian CHI is a breast cancer case-control study conducted in the San Francisco Bay Area, California. The 483 AANHPI women in the CHI study were recruited as controls; details of the sampling and recruitment process have been previously described [17, 18]. In brief, age- and ethnicity- (Chinese, Filipina, other AANHPI) matched controls were recruited through: (1) two community health centers, (2) the Army of Women, a volunteer-based on-line registry, (3) Craigslist and other internet methods, (4) address-based sampling and (5) other community and media outreach efforts. The target number of controls from each source was based on the age and ethnicity distribution of the breast cancer cases enrolled in the full study, and a priori assumptions regarding the distributions of socioeconomic status among controls from each recruitment source. Recruitment of controls was conducted between March 2013 and October 2014.

Survey data were collected contemporaneously from telephone and self-administered interviews and included detailed social and sociodemographic data and data on immigration status, language preference, and lifestyle behaviors. All interviews were conducted in English, Mandarin, Cantonese, or Tagalog. Written informed consent was obtained from all study participants. This research was approved by the Cancer Prevention Institute of California and California Protection for Human Subjects Institutional Review Boards.

### Data collection

#### Social networks

We administered by phone interview a brief egocentric social network questionnaire, which was developed from work in the Indianapolis Network Mental Health Study [19] and adapted from the PhenX Toolkit (https://www.phenxtoolkit.org/protocols/view/211101), to collect data on women’s social networks. The questionnaire employed the following name generator: “Over the past 12 months, who have been the people in your life with whom you discuss important matters? Who are the people you can really count on? These can be people that you see, talk on the phone or via the computer, email, text, or instant message with.” Women provided up to 12 nicknames or sets of initials for these “alters” as well as information about alters’ gender, age, and race/ethnicity and information about the type of relationship (e.g., friend, neighbor, child, grandparent, spouse, member of a religious organization, etc.), level of closeness, and frequency of contact. Women were further asked whether alters knew each other and the level of closeness in those relationships.

From egocentric measures, we computed the degree of each type of relationship. We generated information on degree of relatives from the sum of children, grandchildren, grandparents, in laws, siblings, parents, and other relatives indicated in egocentric analysis and separately evaluated associations with a spouse. We also computed compositional variables (i.e., percent of the total egocentric network comprised of specific types of ties) and analyzed variables with sufficient variation; these included proportions of friends, relatives, females, and NLWs. Since women indicated no Latinx or African-American contacts in their networks, the proportion of NLWs provides the same information as (the inverse of the) proportion of Asians. Density was computed as the number of ties between alters over the total possible number of connections. Most relationships women included in their network were described as very close (or characterized by frequent contact) and so we were only able to examine density of very close relationships or those with frequent contact.

Women were also asked their marital status and whether they were actively involved in any neighborhood groups such as community associations, social clubs, book clubs, churches/spiritual centers, or faith-based organizations. We evaluated associations of these measures and diversity (the number of types of ties) with behavioral outcomes.

#### Other covariates

Available sociodemographic data included age (continuous years), education (continuous years), income (<$24K, 25-35K, 36-45K, 46-55K, 56-65K, 66-75K, 76-99K, 100-149K, 150-199K, 200+K), immigrant status (1^st^, 2^nd^, 3^rd^ generation, <50% of life in the US, ≥50% of life in the US), and language proficiency (speak not at all well to very well). Because English proficiency was correlated with education (r = 0.65, *P*<0.001) and income (r = 0.58, *P*<0.001), because of concerns about collinearity in multiply adjusted analyses, we dichotomized English proficiency (speak very well vs. not). We considered adjusting for internet use and for postmenopausal status, the latter particularly in analyses of waist size, but adjustment for these variables had no substantive effect on associations (S1 Table) and so these covariates were dropped.

#### Lifestyle behaviors, body mass index, and waist size

Smoking history was assessed as ever smoked 100 cigarettes since so few women were current smokers. Participants were asked about hours per week of moderate or strenuous physical activity. Weight and height were self-reported, and waist size and hip size were self-measured based on standardized instructions. BMI in kg/m^2^ was derived from information on weight and height and waist-to-hip ratio (WHR) was computed as waist size divided by hip size. Information on alcohol intake (grams/day) was computed from information on numbers and frequency per week of servings of wine, beer, and liquor. Dietary acculturation questions included in the questionnaire were adapted from the scales developed by Satia et al [20] for Chinese-American women and augmented with data for Filipino [21], Vietnamese [22], Japanese [23], Korean [24–26], and Asian-Indian and Pakistani [27] populations living in the US. We created "Asian" and "Western" scales, assigning 0, 1, or 2 points based on the frequency of consumption of specific items, and summing points. The Western scale included 18 items and the Asian scale 13 items. Asian and Western dietary scales were not a part of the same continuum, but they were inversely correlated (r = -0.47, *P*<0.001).

Continuous health outcome measures included Western diet, Asian diet, drinks per week, hours/week of moderate or strenuous physical activity, BMI, WHR, and waist size. Dichotomous outcomes included those based on median levels of continuous measures (e.g., high Western diet, high Asian diet, physical inactivity); current weight recommendations in studies of AANHPI populations (overweight or BMI≥23 kg/m^2^ [28]; waist size≥85 cm; WHR≥0.85; or any level of consumption for those behaviors with low consumption (any alcohol intake, ever smoked 100 cigarettes). We allowed the sample size to vary depending on the number of participants with data for each behavioral outcome variable. Sample size varied from 445 to 477 participants.

### Statistical analyses

We examined distributions of potential confounding variables, as well as social network characteristics, by the combination of nativity and ethnicity (Chinese, Filipina, other), computing χ^2^ statistics for categorical and F tests for continuous variables.

#### Analyses of social networks and lifestyle factors

We evaluated associations of social network variables (degree, types of ties, diversity of ties, compositional variables, density of very close relationships and those with frequent contact) and continuous outcomes (body mass index (BMI), waist size, waist to hip ratio (WHR), Asian diet, Western diet, drinks per week) using linear regression (PROC GENMOD, SAS Institute, Cary, NC). We used multivariable logistic regression (PROC LOGISTIC) to estimate odds ratios (ORs) and 95% confidence intervals (CI) for analyses of dichotomous outcome variables (<8 hours moderate or strenuous activity/week, BMI≥23 kg/m2, WHR≥0.85, ever smoked 100 cigarettes, any alcohol intake). Because of limited numbers of women specifically indicating colleagues, professionals, neighbors, or members of a religious organization as alters within their social networks, we dropped these variables from analyses. We evaluated associations adjusted for age only and subsequently for multiple covariates including age, ethnicity, education, income, immigration status, and high English-speaking proficiency.

## Results

Of the 477 women in the study, 51% were Chinese, 21% were Filipina, and 28% were of other ethnicities (Table 1). Women were 22–82 years of age (mean and median = 50). Thirty-five percent were born in the US; of those who immigrated to the US, 25% immigrated before age 20. Forty-eight percent of women were postmenopausal. Eight percent had a high school education or less, an additional 20% obtained some college education, and the great majority (72%) obtained post-graduate education. Fifty-nine percent indicated they spoke English very well. Examining by nativity (US- vs. foreign-born) and ethnicity, Chinese and Filipina women were similar in age when they immigrated, but US-born Filipina women in the study were younger than Chinese women or foreign-born Filipina women. Foreign-born women had slightly less education than US-born women and felt less comfortable with their English skills than women born in the US.

**Table 1:** Selected characteristics by nativity and ethnicity (N = 477).

		US-born			Foreign-born			
	All	Chinese	Filipina	Other ethnicity	Chinese	Filipina	Other ethnicity	*P*^c^
N	477	68	21	77	177	78	56	
Age (mean, SD)	50.5 (11.7)	50.3 (10.5)	39.7 (11.4)	47.3 (12.7)	53.7 (11.6)	51.7 (10.7)	47.6 (9.2)	<0.0001
Age at immigration^a^ (mean, SD)	30.3 (15.8)	N/A	N/A	N/A	31.9 (15.0)	34.7 (16.5)	19.2 (12.2)	<0.0001
Postmenopausal (N, %)	228 (48.0)	30 (44.1)	5 (23.8)	30 (39)	95 (53.7)	49 (62.8)	19 (35.2)	0.0009
Education (N, %)								<0.0001
High school or less	38 (8.0)	0 (0.0)	0 (0.0)	0 (0.0)	33 (18.6)	3 (3.0)	2 (3.6)	
College	92 (19.5)	5 (7.4)	3 (14.3)	18 (23.4)	41 (23.2)	23 (29.9)	3 (5.4)	
Post-graduate	345 (72.5)	63 (92.7)	18 (85.7)	59 (76.6)	103 (58.2)	51 (66.2)	51 (91.1)	
Income^b^ (N, %)								<0.0001
$35,000 or less	117 (29.3)	5 (8.1)	3 (14.3)	14 (19.4)	76 (47.2)	13 (34.2)	6 (13.0)	
$36,000-$99,000	120 (30.0)	20 (32.3)	10 (47.6)	21 (29.2)	40 (24.8)	14 (36.8)	15 (32.6)	
$100,000 or more	163 (40.8)	37 (59.7)	8 (38.1)	37 (38.1)	45 (28.0)	11 (29.0)	25 (54.4)	
High English proficiency (N, %)	280 (58.7)	67 (98.5)	21 (100)	77 (100)	37 (20.9)	39 (50.0)	39 (69.6)	<0.0001

Abbreviations: SD, standard deviation

^a^Among women not born in the US

^b^Among those who provided information on income

^c^p-value, χ^2^ test for categorical variables and F test for continuous variables

Social network variables differed across ethnicity and nativity (Table 2). US-born AANHPI women indicated larger networks than did foreign-born women. They also had substantially higher proportions of NLWs in their networks. Women from different ethnic/nativity groups did not differ with regard to community participation, high density of relationships with frequent contact, or proportion of friends in their networks. By contrast, foreign-born women were slightly more likely to be married. Foreign-born women had higher proportions of relatives in their networks compared with ethnically similar US-born women. Regardless of nativity, Filipina women had the highest proportion of relatives in their networks; foreign-born Filipina women had the highest density of very close relationships.

**Table 2:** Egocentric social network characteristics of Asian American, Native Hawaiian, and Pacific Islander women from the Asian Community Health Initiative Study (N = 477).

		US-born			Foreign-born			
	All	Chinese	Filipina	Other ethnicity	Chinese	Filipina	Other ethnicity	*P*^a^
N	477	68	21	77	177	78	56	
Degree (Mean, SD)								
All	3.8 (1.8)	4.1 (2.0)	4.9 (1.9)	4.1 (1.5)	3.6 (1.9)	3.7 (1.6)	3.3 (1.7)	0.002
Relatives	1.7 (1.3)	1.5 (1.1)	2.4 (1.4)	1.6 (1.3)	1.7 (1.2)	2.1 (1.4)	1.5 (1.5)	0.002
Friends	1.3 (1.4)	1.9 (1.8)	1.7 (1.7)	1.5 (1.3)	1.1 (1.4)	1.1 (1.2)	1.1 (1.1)	0.0009
Married (%)	66.5	63.2	42.9	59.7	70.1	66.7	76.8	0.05
Community participation (%)	2.7	4.4	4.8	3.9	1.1	3.8	1.8	0.59
Composition (%)								
Relatives	45.7	38.3	52.1	37.1	47.6	58.4	40.6	<0.0001
Friends	32.2	39.7	30.9	38.4	28.9	27.8	32.8	0.08
Non-Latinx White	15.2	27.1	21.6	34.3	6.3	1.6	19.5	<0.0001
High density (%)								
Very close relationships	64.6	63.2	66.7	68.8	57.6	82.1	57.1	0.006
Frequent contact	53.0	45.6	47.6	41.6	55.4	61.5	60.7	0.08

Abbreviations: SD, standard deviation

^a^p-value, F-test

### Social networks and continuous outcomes

In minimally (S2 Table) and multivariable-adjusted analyses (Table 3), higher degree and/or proportion of relatives were associated with higher BMI and higher waist size. They were also related to higher Asian and lower Western diet in minimally-adjusted analyses though associations were diminished with multiple adjustment. Conversely, higher degree and/or proportion of friends were associated with lower BMI and waist size and higher Western diet. A higher percentage of NLW in women’s networks was associated with lower Asian diet, higher Western diet, and lower waist size and WHR. Being married was associated with both higher Asian diet and lower alcohol intake. A high density of relationships with frequent contact was associated with higher Asian and lower Western diet. By contrast, community participation was associated with lower Asian and higher Western diet. Neither diversity of ties nor percent of females in women’s networks was related to behavioral outcomes.

**Table 3:** Social network variables and multivariable-adjusted linear associations^a^ with behavioral risk factors (N = 477).

	BMI (kg/m2)	*P*^b^	Waist size	*P*^b^	WHR	*P*^b^	Asian diet	*P*^b^	Western diet	*P*^b^	Drinks per week	*P*^b^
Degree^c^												
All	0.09	0.44	0.02	0.84	0.0004	0.83	0.13	0.15	0.09	0.53	-0.02	0.75
Relatives	0.41	0.01	0.34	0.04	0.003	0.32	0.23	0.06	-0.26	0.17	-0.02	0.77
Friends	-0.26	0.09	-0.28	0.08	-0.003	0.31	-0.08	0.51	0.38	0.03	0.05	0.54
Married	0.65	0.19	0.75	0.12	0.01	0.11	1.22	0.0007	0.33	0.54	-0.54	0.03
Community participation	-1.32	0.34	-1.39	0.29	-0.04	0.07	-2.27	0.02	3.20	0.03	-0.18	0.79
Composition (%)												
Relatives	1.37	0.05	1.45	0.05	0.004	0.73	0.57	0.29	-1.43	0.08	-0.09	0.81
Friends	-1.39	0.03	-1.51	0.02	-0.01	0.31	-0.70	0.16	1.47	0.05	0.31	0.36
Non-Latinx White	-1.13	0.20	-2.55	0.004	-0.03	0.04	-2.75	< .0001	1.68	0.09	0.31	0.48
High density												
Very close relationships	0.50	0.27	0.69	0.13	0.004	0.57	0.62	0.07	-0.68	0.18	0.13	0.58
Frequent contact	-0.004	0.99	0.26	0.54	0.009	0.19	1.10	0.0005	-0.90	0.06	-0.09	0.69

Abbreviations: BMI, body mass index; WHR, waist-to-hip ratio

^a^Models adjusted for age, ethnicity, education, income, immigration status, and high English proficiency.

^b^p-value, Wald test

^c^Associations with hours of moderate or strenuous activity not statistically significant. Percent females and diversity of ties unrelated to behavioral risk factors

### Social networks and dichotomous lifestyle factors

Findings from minimally-adjusted (S3 Table) and multivariable-adjusted (Table 4) analyses were similar for dichotomous outcomes. In multivariable-adjusted analyses, degree or social network size was not associated with lifestyle behaviors. However, a higher degree of relatives was related to higher odds of BMI≥23 (OR = 1.19, 95% CI:1.02–1.38) and of ever smoking 100 cigarettes (OR = 1.28, 95% CI:1.02–1.60). Similarly, a higher proportion of relatives in women’s networks was related to higher odds of BMI≥23 (OR = 2.15, 95% CI:1.12–4.12) and of ever smoking 100 cigarettes (OR = 3.02, 95% CI:1.04–8.76). By contrast, a higher degree of friends was related to a lower likelihood of <8 hours physical activity per week (OR = 0.86, 95% CI:0.74–1.00), a lower likelihood of BMI≥23 (OR = 0.83, 95% CI:0.71–0.96), and a higher likelihood of alcohol intake (OR = 1.18, 95% CI:1.01–1.39). Any community participation was associated with a lower likelihood of WHR≥0.85 (OR = 0.12, 95% CI:0.03–0.62). Neither high density of very close relationships nor those with frequent contact was related to outcomes.

**Table 4:** Social network variables and multivariable-adjusted relative odds^a^ of behavioral risk factors (N = 477).

	<8 hrs moderate or strenuous activity/wk	95% CI	BMI ≥23 kg/m2	95% CI	WHR> = 0.85^b^	95% CI	Ever smoked 100 cigarettes	95% CI	Any alcohol intake	95% CI
Degree^c^										
All	0.93	(0.83, 1.04)	0.96	(0.86, 1.07)	0.99	(0.88, 1.10)	1.05	(0.89, 1.25)	1.07	(0.95, 1.21)
Relatives	1.01	(0.87, 1.17)	1.19	(1.02, 1.38)	1.00	(0.85, 1.17)	1.28	(1.02, 1.60)	0.97	(0.82, 1.14)
Friends	0.86	(0.74, 1.00)	0.83	(0.71, 0.96)	0.98	(0.85, 1.13)	0.86	(0.68, 1.09)	1.18	(1.01, 1.39)
Married	1.51	(0.97, 2.37)	1.21	(0.78, 1.89)	1.25	(0.78, 1.98)	1.04	(0.53, 2.04)	0.75	(0.47, 1.22)
Community participation	1.04	(0.32, 3.43)	0.64	(0.18, 2.27)	0.12	(0.03, 0.62)	1.60	(0.38, 6.74)	0.65	(0.18, 2.35)
Composition (%)										
Relatives	1.37	(0.71, 2.65)	2.15	(1.12, 4.12)	0.99	(0.49, 1.99)	3.02	(1.04, 8.76)	0.68	(0.34, 1.40)
Friends	0.70	(0.38, 1.29)	0.59	(0.32, 1.07)	0.92	(0.49, 1.72)	0.46	(0.17, 1.25)	1.34	(0.70, 2.58)
Non-Latinx White	1.44	(0.65, 3.21)	0.76	(0.34, 1.69)	0.33	(0.14, 0.76)	1.55	(0.55, 4.34)	1.23	(0.50, 3.03)
High density										
Very close	1.11	(0.74, 1.67)	1.07	(0.71, 1.62)	1.35	(0.88, 2.06)	0.92	(0.49, 1.75)	0.97	(0.62, 1.51)
Frequent contact	1.30	(0.88, 1.93)	0.93	(0.63, 1.37)	1.38	(0.92, 2.07)	1.24	(0.68, 2.27)	0.74	(0.49, 1.13)

Abbreviations: BMI, body mass index; CI, confidence interval; WHR, waist-to-hip ratio

^a^Models adjusted for age, ethnicity, education, income, immigration status, and high English proficiency.

^b^Associations with odds of waist≥32 inches similar to associations with WHR≥0.85.

^c^Odds ratios with Western diet not statistically significant. Percent females and diversity of ties unrelated to behavioral risk factors

A higher proportion of NLWs in women’s networks was related to a lower likelihood of WHR≥0.85 (OR = 0.33, 95% CI:0.14–0.76). Multivariable adjustment attenuated the association of NLW with both smoking and alcohol. Being married was related to a slightly higher odds of <8 hours physical activity per week (OR = 1.51, 95% CI:0.97–2.37). Neither the proportion of females in women’s networks nor diversity of ties were related to behavioral outcomes.

## Discussion

Characteristics of social networks including degree, composition, and density as well as types of ties were related to lifestyle behaviors among AANHPI women. Women with networks with a higher degree and composition of friends and NLWs had lower BMI and waist size, lower Asian diets, and higher Western diets. Community participation was also associated with lower Asian and higher Western diet. Conversely, higher degree and proportion of relatives were associated with higher BMI and waist size, lower Western diet, and higher odds of ever smoking. Similarly, a high density of relationships characterized by frequent contact, was also associated with higher Asian and lower Western diet. Being married was associated with higher Asian diet and lower alcohol consumption. This is the first study examining egocentric social network characteristics and lifestyle behaviors in a US-living AANHPI population of women.

Previous work has demonstrated that social networks influence lifestyle factors both in general (e.g., physical activity [29]; diet [13, 30]; alcohol [8, 31]; obesity [14]) and in Asian populations (e.g., diet [32]; physical activity [33]; smoking [34, 35]). More specifically, large, diverse social networks and family and friends who model or support behaviors have been consistently related to healthier lifestyle behaviors, mediated by social norms [30, 36, 37], social support [38], and seeking of social activity [33]. Investigators have used varied social network measures including epidemiologic and egocentric social network measures. However, exceedingly limited work has been conducted in AANHPI women specifically and most prior work has focused on single behavioral outcomes, limiting the ability to examine patterns of associations more broadly.

Associations in this study were consistent with social influences associated with Western norms on behaviors including alcohol intake, smoking, and diet and thus showed parallels to the literature on acculturation and smoking [39–41], diet [41–43], and physical activity [42, 44]. Women who have been in the US longer often have a higher Western diet intake, particularly higher intakes of meat, sweets, and sweetened beverages, a greater likelihood of smoking, and higher levels of physical activity [45–47]. In prior work, social network composition has differed by nativity with immigrant (vs. US-born) women having larger proportions of family in their networks [48, 49]. Findings here suggest that adoption of Western lifestyle behaviors may in part occur through networks of friends and of NLWs whereas family networks and AANHPI networks were associated with greater retention of behaviors more typical of countries of origin.

Though Western behaviors associated with greater acculturation have generally been associated with greater obesity [42, 43, 50, 51], women with more “Western” social networks in our study, i.e., those characterized by greater proportions of NLWs and friends, had lower BMI and waist size. Lifestyle behaviors may be patterned by Asian sociocultural norms for thinness [52, 53] as well as Western norms for thinness associated with higher socioeconomic status [54, 55], consistent with the high levels of education in this cohort.

Though the influence of social networks on lifestyle behaviors may reflect acculturation, social networks had independent influences on behavioral outcomes adjusted for measures of acculturation including nativity, English proficiency, and years in the US. Consistent with this, in a study of 591 Korean women [56], having more ‘encouragers’ and fewer ‘discouragers’ of alcohol intake in women’s social network, not acculturation assessed using an acculturative identity scale, was related to a higher likelihood of drinking. In another study of 766 South Asians, 44.1% of whom were women, perceptions of body size among the five closest persons in participants’ social networks, were positively related to body size norms and self-assessed body size [36] apart from cultural identity or years in the US.

A major strength of the current analysis was the ability to examine associations with a variety of social network characteristics, assessed through a well-established, publicly available egocentric social network data tool. This study is the first to examine associations with lifestyle behaviors in an ethnically diverse population of AANHPI women. In addition, we were able to adjust for variables including socioeconomic status, immigration status, and language proficiency, that may constrain social networks and also influence lifestyle behaviors. An additional strength was the extensive set of detailed data on lifestyle behaviors.

A major limitation was the lack of power to examine associations by country of origin and to examine intersectional influences of country of origin, socioeconomic status, and social network variables on lifestyle behaviors [57]. Future, larger studies should consider these influences given substantial ethnic group heterogeneity [58, 59]; larger studies are needed generally. Our results may not generalize to women of lower education who were not well represented in this population. Examining associations in a more representative population may lead to different associations, particularly those with body weight and waist size. Future studies should ideally include a larger population with greater socioeconomic and ethnic diversity.

It is not possible to fully interpret variation in the number of alters women provided. Though this appeared to be influenced by the numbers of people women described as close, factors that may also influence the number of alters provided include conscientiousness, time constraints, and interest in the question, in addition to the numbers of important persons in a woman’s network. Another limitation, associations were cross-sectional. Rather than representing the influence of social network influences on behaviors, homophily, or association with like others, could lead to apparent associations with behaviors if shared behaviors increase the likelihood that people associate with each other. Furthermore, poor health resulting from adverse lifestyle can lead to greater social isolation or influence network composition [60–62]. Nonetheless, this didn’t help to explain why greater friendship networks, and smaller relative networks, were related to lower body weight, waist size, and a lower likelihood of overweight in our sample. Rather, patterns were consistent with the influence of social networks and related social norms associated with living in the US, with diverging influences of friends and family on behaviors. Future work should examine longitudinal changes in networks and lifestyle behaviors.

To summarize, our findings provide evidence for the influence of the social environment, as measured by the size, composition, and structure of women’s social networks, on lifestyle behaviors in AANHPI women. More specifically and most notably, larger proportions of friends and NLWs in the social networks of AANHPI women were related to lower BMI and waist size, lower Asian diet, and higher Western diet. Conversely, a larger proportion of relatives was associated with higher BMI and waist size, lower Western diet, and higher odds of ever smoking. These findings have implications for disease risk and preventative social network interventions.

## Supporting information

S1 Table Social network variables and multiply-adjusted^a^ linear associations with behavioral risk factors (N = 477). Abbreviations: BMI, body mass index; WHR, waist-to-hip ratio.
(DOCX)

S2 Table Social network variables and minimally-adjusted^a^ linear associations with behavioral risk factors (N = 477). Abbreviations: BMI, body mass index; WHR, waist-to-hip ratio.
(DOCX)

S3 Table Social network variables and minimally-adjusted^a^ relative odds of behavioral risk factors (N = 477). Abbreviations: BMI, body mass index; CI, confidence interval; WHR, waist-to-hip ratio.
(DOCX)

## Abbreviations

AANHPI: Asian American, Native Hawaiian, and Pacific Islander
BMI: body mass index
CHI: Asian Community Health Initiative
CI: confidence interval
NLW: non-Latinx Whites
OR: odds ratio
SD: standard deviation
WHR: waist-to-hip ratio
