# Supplementary material for: Egocentric social networks, lifestyle behaviors, and body size in the Asian Community Health Initiative (CHI) cohort
Source: PLoS One. 2020 May 6;15(5):e0232239. doi: 10.1371/journal.pone.0232239 (PMC7202641; doi:10.1371/journal.pone.0232239)
Supplement: S1 Table — Abbreviations: BMI, body mass index; WHR, waist-to-hip ratio. (DOCX) [file pone.0232239.s001.docx]

|  | BMI (kg/m2) | *P*^b^ | Waist size | *P*^b^ | WHR | *P*^b^ | Asian diet | *P*^b^ | Western diet | *P*^b^ | Drinks/week | *P*^b^ |
| --- | --- | --- | --- | --- | --- | --- | --- | --- | --- | --- | --- | --- |
| Degree^c^ |  |  |  |  |  |  |  |  |  |  |  |  |
| All | 0.09 | 0.45 | 0.02 | 0.89 | 0.000 | 0.86 | 0.15 | 0.10 | 0.10 | 0.49 | -0.02 | 0.72 |
| Relatives | 0.39 | 0.02 | 0.33 | 0.05 | 0.003 | 0.35 | 0.25 | 0.04 | -0.25 | 0.17 | -0.03 | 0.75 |
| Friends | -0.25 | 0.11 | -0.28 | 0.08 | -0.002 | 0.36 | -0.09 | 0.46 | 0.38 | 0.03 | 0.05 | 0.53 |
| Married | 0.69 | 0.16 | 0.80 | 0.10 | 0.01 | 0.08 | 1.16 | 0.001 | 0.36 | 0.51 | -0.53 | 0.03 |
| Community participation | -1.45 | 0.29 | -1.50 | 0.25 | -0.04 | 0.06 | -2.24 | 0.03 | 3.09 | 0.03 | -0.23 | 0.72 |
| Composition (%) |  |  |  |  |  |  |  |  |  |  |  |  |
| Relatives | 1.30 | 0.07 | 1.39 | 0.06 | 0.003 | 0.80 | 0.59 | 0.28 | -1.45 | 0.07 | -0.09 | 0.80 |
| Friends | -1.28 | 0.05 | -1.44 | 0.03 | -0.009 | 0.41 | -0.90 | 0.07 | 1.48 | 0.05 | 0.35 | 0.30 |
| Non-Latino/a White | -1.28 | 0.15 | -2.70 | 0.003 | -0.03 | 0.02 | -2.61 | <.0001 | 1.66 | 0.10 | 0.27 | 0.54 |
| High density |  |  |  |  |  |  |  |  |  |  |  |  |
| Very close relationships | 0.53 | 0.24 | 0.74 | 0.10 | 0.005 | 0.49 | 0.57 | 0.09 | -0.65 | 0.20 | 0.15 | 0.50 |
| Frequent contact | 0.05 | 0.90 | 0.39 | 0.37 | 0.01 | 0.14 | 1.04 | 0.001 | -0.86 | 0.08 | -0.04 | 0.87 |

^a^Models adjusted for age and ethnicity, education, income, immigration status, high English proficiency, internet use, and menopausal status

^b^p-value, Wald test

^c^Associations with hours of moderate or strenuous activity were not statistically significant. Percent females unrelated to behavioral risk factors
