# Supplementary material for: Egocentric social networks, lifestyle behaviors, and body size in the Asian Community Health Initiative (CHI) cohort
Source: PLoS One. 2020 May 6;15(5):e0232239. doi: 10.1371/journal.pone.0232239 (PMC7202641; doi:10.1371/journal.pone.0232239)
Supplement: S2 Table — Abbreviations: BMI, body mass index; WHR, waist-to-hip ratio. (DOCX) [file pone.0232239.s002.docx]

|  | BMI (kg/m2) | *P*^b^ | Waist size | *P*^b^ | WHR | *P*^b^ | Asian diet | *P*^b^ | Western diet | *P*^b^ | Drinks/week | *P*^b^ |
| --- | --- | --- | --- | --- | --- | --- | --- | --- | --- | --- | --- | --- |
| Degree^c^ |  |  |  |  |  |  |  |  |  |  |  |  |
| All | 0.05 | 0.70 | -0.02 | 0.85 | -0.001 | 0.57 | -0.11 | 0.32 | 0.35 | 0.02 | 0.04 | 0.50 |
| Relatives | 0.47 | 0.005 | 0.40 | 0.02 | 0.004 | 0.19 | 0.28 | 0.06 | -0.32 | 0.12 | -0.03 | 0.70 |
| Friends | -0.29 | 0.06 | -0.31 | 0.05 | -0.005 | 0.08 | -0.40 | 0.003 | 0.72 | 0.0001 | 0.13 | 0.11 |
| Married | -0.07 | 0.89 | 0.12 | 0.80 | 0.007 | 0.39 | 1.59 | <.0001 | -0.19 | 0.74 | -0.68 | 0.004 |
| Community participation | -0.67 | 0.64 | -1.14 | 0.40 | -0.04 | 0.07 | -3.52 | 0.002 | 4.58 | 0.005 | 0.24 | 0.72 |
| Composition (%) |  |  |  |  |  |  |  |  |  |  |  |  |
| Relatives | 1.86 | 0.009 | 1.92 | 0.008 | 0.01 | 0.25 | 1.44 | 0.02 | -2.44 | 0.006 | -0.28 | 0.45 |
| Friends | -1.41 | 0.04 | -1.54 | 0.02 | -0.01 | 0.19 | -1.35 | 0.02 | 2.20 | 0.008 | 0.48 | 0.16 |
| Non-Latino/a White | -0.64 | 0.45 | -2.20 | 0.009 | -0.04 | 0.007 | -5.78 | <.0001 | 5.41 | <.0001 | 1.10 | 0.009 |
| High density |  |  |  |  |  |  |  |  |  |  |  |  |
| Very close relationships | 0.58 | 0.21 | 0.68 | 0.14 | 0.003 | 0.66 | 0.33 | 0.42 | -0.34 | 0.55 | 0.19 | 0.43 |
| Frequent contact | -0.07 | 0.88 | 0.21 | 0.63 | 0.01 | 0.17 | 1.38 | 0.0003 | -1.24 | 0.02 | -0.19 | 0.39 |

^a^Models adjusted for age and ethnicity

^b^p-value, Wald test

^c^Associations with hours of moderate or strenuous activity were not statistically significant. Percent females unrelated to behavioral risk factors
