# Supplementary material for: Egocentric social networks, lifestyle behaviors, and body size in the Asian Community Health Initiative (CHI) cohort
Source: PLoS One. 2020 May 6;15(5):e0232239. doi: 10.1371/journal.pone.0232239 (PMC7202641; doi:10.1371/journal.pone.0232239)
Supplement: S3 Table — Abbreviations: BMI, body mass index; CI, confidence interval; WHR, waist-to-hip ratio. (DOCX) [file pone.0232239.s003.docx]

|  | <8 hrs activity/wk | 95% CI | BMI>23 kg/m2 | 95% CI | WHR>0.85^b^ | 95% CI | Ever smoked 100 cigarettes | 95% CI | Any alcohol intake | 95% CI |
| --- | --- | --- | --- | --- | --- | --- | --- | --- | --- | --- |
| Degree^c^ |  |  |  |  |  |  |  |  |  |  |
| All | 0.92 | (0.83, 1.02) | 0.95 | (0.85, 1.05) | 0.95 | (0.85, 1.05) | 1.14 | (0.97, 1.32) | 1.15 | (1.03, 1.30) |
| Relatives | 1.00 | (0.86, 1.16) | 1.20 | (1.03, 1.39) | 1.02 | (0.88, 1.19) | 1.23 | (1.00, 1.51) | 0.94 | (0.81, 1.10) |
| Friends | 0.85 | (0.74, 0.98) | 0.83 | (0.72, 0.95) | 0.93 | (0.81, 1.06) | 1.00 | (0.81, 1.24) | 1.30 | (1.11, 1.51) |
| Married | 1.51 | (1.00, 2.27) | 1.01 | (0.67, 1.52) | 1.09 | (0.71, 1.66) | 0.76 | (0.42, 1.38) | 0.76 | (0.50, 1.16) |
| Community participation | 0.90 | (0.28, 2.90) | 0.80 | (0.24, 2.70) | 0.13 | (0.03, 0.61) | 2.20 | (0.55, 8.75) | 1.02 | (0.30, 3.48) |
| Composition (%) |  |  |  |  |  |  |  |  |  |  |
| Relatives | 1.33 | (0.71, 2.51) | 2.41 | (1.29, 4.50) | 1.28 | (0.66, 2.48) | 1.96 | (0.76, 5.06) | 0.49 | (0.25, 0.96) |
| Friends | 0.66 | (0.37, 1.20) | 0.60 | (0.33, 1.06) | 0.84 | (0.46, 1.54) | 0.70 | (0.28, 1.74) | 1.65 | (0.89, 3.06) |
| Non-Latino/a White | 0.95 | (0.46, 1.97) | 0.85 | (0.42, 1.73) | 0.28 | (0.13, 0.61) | 3.16 | (1.22, 8.18) | 3.13 | (1.35, 7.24) |
| High density |  |  |  |  |  |  |  |  |  |  |
| Very close | 1.06 | (0.71, 1.58) | 1.10 | (0.74, 1.64) | 1.29 | (0.85, 1.95) | 0.93 | (0.50, 1.72) | 1.04 | (0.69, 1.58) |
| Frequent contact | 1.40 | (0.96, 2.05) | 0.90 | (0.62, 1.32) | 1.40 | (0.94, 2.07) | 0.99 | (0.56, 1.76) | 0.68 | (0.46, 1.01) |

^a^Models adjusted for age and ethnicity.

^b^Associations with odds of waist>32 inches similar to associations with WHR>0.85.

^c^Odds ratios with > median Western diet not statistically significant. Percent females unrelated to behavioral risk factors
